# Long Non-Coding RNA H19 Positively Associates With Aspirin Resistance in the Patients of Cerebral Ischemic Stroke

**DOI:** 10.3389/fphar.2020.580783

**Published:** 2020-09-25

**Authors:** Jue Wang, Bin Cao, Yan Gao, Dong Han, Haiping Zhao, Yuhua Chen, Yumin Luo, Juan Feng, Yanxia Guo

**Affiliations:** ^1^ Department of Neurology, Shengjing Hospital of China Medical University, Shenyang, China; ^2^ Institute of Cerebrovascular Diseases Research and Department of Neurology, Xuanwu Hospital of Capital Medical University, Shenyang, China; ^3^ Department of Developmental Cell Biology, Key Laboratory of Cell Biology, Ministry of Public Health, and Key Laboratory of Medical Cell Biology, Ministry of Education, China Medical University, Shenyang, China

**Keywords:** H19, aspirin resistance (AR), long non-coding RNA, 11dhTXB2, recurrent stroke

## Abstract

**Background and purpose:**

Aspirin is a novel anti-platelet drug that is intensively recommended for the prevention and treatment of cerebral ischemic stroke. However, the existence of aspirin resistance weakens the effects of aspirin and usually induces the recurrence of ischemic stroke. While the mechanism underlying aspirin resistance is still unclear. Long non-coding RNA H19 (H19) is closely associated with the onset and prognosis of cerebral ischemic stroke. Since the relationship between H19 and aspirin resistance have never been reported, herein, we aimed to evaluate the H19 expression in aspirin-resistant ischemic stroke patients and subsequently, ascertain the ability of H19 to diagnose aspirin resistance.

**Methods:**

We included 150 patients with acute cerebral ischemic stroke who were followed up for one year to determine stroke recurrence. Levels of 11-dehydro thromboxane B2 (11dhTXB2) in urine were tested to evaluate the status of aspirin resistance, and those of H19 and 8-iso-prostaglandin-2α in plasma were assessed. The relationship between 11dhTXB2 or and 8-iso-prostaglandin-2α and H19, and the receiver operating characteristic curve of H19, the association of H19 and aspirin resistance with the recurrence of stoke were statistically analyzed.

**Results:**

Plasma H19 was significantly up-regulated in patients with aspirin resistance (p=0.0203), and the H19 levels were positively associated with urine 11dhTXB2/creatinine (R=0.04364, p=0.0106) and positively associated with the level of 8-iso-PGF2α (R=0.04561, p=0.0089). The ROC curves indicated that H19 can sensitively and specifically diagnose aspirin resistance (area under the curve, 0.8005; 95% CI, 0.7301–0.8710; p < 0.0001; specificity, 75.86207%; sensitivity, 73.84615%.). H19 is an independent risk factor for aspirin resistance (OR=1.129, p=0.0321), and aspirin resistance and H19 are closely related with ischemic stroke recurrence.

**Conclusions:**

H19 is closely associated with aspirin resistance, and H19 probably induces aspirin resistance through increasing the production of 8-iso-prostaglandin-2α. Besides which, H19 may serve as a serological marker for diagnosing aspirin resistance with high specificity and sensitivity, and the test of H19 could give clues to the recurrence of ischemic stroke.

## Introduction

Aspirin is a novel anti-platelet drug. A low dose of oral aspirin (100–200 mg) is used to prevent and treat cerebral ischemic stroke, which is recommended by the “2018 Guidelines for the Early Management of Patients with Acute Ischemic Stroke” (Powers et al., 2018). Its low costs and convenient administration encourage good patient compliance. However, routine aspirin medication cannot always prevent and treat ischemic stroke (Yi et al., 2016; Wang et al., 2018). The reason for this is the low physiological responsiveness to aspirin, which diminishes its anti-platelet effects and results in a failure to prevent atherosclerotic events; this phenomenon is termed aspirin resistance (Al-Jabi Samah, 2017).

One report has indicated that 5% to 45% of the population has aspirin resistanve (Mason Peter et al., 2005). Patients with aspirin resistance always develop recurrent ischemic stroke, and aspirin resistance is positively associated with the severity and the infarction volume of such strokes (Yi et al., 2016; Cheng et al., 2017). Low dose aspirin inhibits cyclooxygenase-1 (COX-1) activity to decrease thromboxane A2 (TXA2) production and induce anti-platelet effects (Lopez et al., 2014; McCullough et al., 2017; Chen and Chou, 2018). Any substances that interfere with COX-1 and TXA2 production or activity would influence the effects of aspirin. The mechanism underlying AR is uncertain, but oxidative stress might be involved in this process (Guo et al., 2019).

The H19 gene is maternal expressed and paternally imprinted. Mutations in its single nucleotide polymorphism are associated with the onset and progression of gastric cancer, coronary artery diseases, obesity and hypertension (Hernandez-Valero et al., 2013; Tragante et al., 2014; Gao et al., 2015; Yang et al., 2015). We recently showed that H19 polymorphisms increase susceptibility to ischemic stroke (Wang et al., 2017a). The long non-coding RNA H19 (H19) is transcribed form the H19 gene. It has been investigated in detail. We previously showed that H19 participates in the onset and prognosis of cerebral ischemic stroke by regulating neuronal autophagy, microglial polarization and neurogenesis (Wang et al., 2017a; Wang et al., 2017b; Wang et al., 2019).

Since H19 has been reported to be participated in the process of oxidative stress (Yu et al., 2019) and oxidative stress is a common cause of aspirin resistance, we hypothesize that H19 takes part in the production of aspirin resistance. The relationship between H19 and COX-1 or TXA2 has not been reported. Oxidative stress may be the connection between H19 and aspirin resistance. It has been reported that the up-regulation of H19 would lead to the accumulation of ROS and cause serious oxidative stress (Yu et al., 2019), and the overload of oxidative stress could activate 8-iso-prostaglandin-2α (8-iso-PGF2α) induced cell signal transduction pathway and finally result in aspirin resistance (Patrignani, 2003; Guo et al., 2019).

Thus, with the aim of underscoring the significance of H19 in ischemic stroke, in the present study, we explored the differences in H19 expression between i aspirin- sensitive and aspirin-resistance patients, analyzed the ability of H19 to diagnose aspirin resistance, and attempted to elucidate the mechanism underlying aspirin resistance.

## Materials and Methods

### The Collection of Clinical Data

We recruited 150 patients with acute ischemic stroke, from Shengjing Hospital, the affiliated hospital of China Medical University, between December 1, 2017 and December 31, 2018. All patients presented at hospital with sudden onset, partial, or global cerebral function loss, with the formation of ischemic foci in related cerebral functional regions confirmed by magnetic resonance imaging (MRI). The patients had no history of cerebral ischemic stroke or routine aspirin medication. The severity of neurological deficits was examined immediately based on the National Institutes Health Stoke Scale (NIHSS) scores. Neurologists who were blinded to the condition of the patients performed the diagnostic tests and NIHSS evaluations. The aims and protocols of the study were explained to the patients and they were assured that their privacy rights were protected. The patients provided written informed consent to participate in the study, which was approved by the Ethics Committee of the Shengjing Hospital and China Medical University. The blood and urine of the patients were collected after their administration, and stored at −80^°^C after centrifugation before tested together. All the patients included in this study were induced routine admission examination, including blood routine, hepatic and renal function, blood lipid series and so on. Their basic information and previous medical history were collected.

### Determination of Aspirin Resistance

The urine sample of the patients was collected, centrifugated to exclude the sediments and stored at −80^°^C. Urine levels of 11dhTXA2 were measured using ELISA kits ((Cat# TXL-036; RRID: AB_2819213; Corgenix Medical Corp., Broomfield, CO, USA) as per manufacturer’s instructions. Besides which, the level of creatinine in the urine was also tested. The status of aspirin responsiveness was determined based on the urinary 11dhTXA2/creatinine values; values > 1500 pg/mg indicated aspirin resistance, and values < 1500 pg/mg indicated aspirin sensitivity.

### The Test of Plasma H19 Level

The blood sample of the patients was collected in the test tube with anti-coagulation solution, centrifugated to get the plasma and stored at −80^°^C. Plasma H19 levels were analyzed by real-time PCR. Briefly, SYBR Green qPCR Master Mix (Fermentas Canada Inc., Burlington, ON, Canada) was used to amplify the cDNA on a StepOne sequence detection system (Applied Biosystems, Foster City, CA, USA). The primers used to determine H19 levels were as follows: 5′-GAA GGC CAA GAC GCC AGG-3′ (forward) and 5′-TCC TCT GTC CTC GCC GTC AC-3′ (reverse). Levels of H19 were normalized as fold change relative to that of β-actin. The primers used to detect of β-actin levels were: 5′-GTG GCC GAG GAC TTT GAT TG-3′ (forward) and 5′-CCT GTA ACA ACG CAT CTC ATA TT-3′ (reverse).

### Determination of Recurrent Ischemic Stroke

Recurrent ischemic stroke was followed up by applying a standard questionnaire *via* telephone calls every 3 months for one year after the patients were discharged from hospital. The endpoint of the follow-up was the recurrence of ischemic stroke recurrence. The recurrence of ischemic stroke was defined as sudden functional deterioration with a decrease in NIHSS score of ≥ 4, or a new neurological deficit induced by vascular original cerebral foci lasting longer than 24 h.

### Statistical Analysis

All data are expressed as the mean value ± SD. Plasma H19 expression and urinary 11dhTXA2/creatinine values were analyzed using t-tests. Values with P < 0.05 were considered statistically significant. All the categorical variables were compared using chi-square tests. The Hardy-Weinberg equilibrium was evaluated using chi-square goodness-of–fit tests. Aspirin resistance was predicted by constructing receiver operating characteristic (ROC) curves for H19.

## Results

### Basic Information About the Patients

We separated 150 patients into the AR and AS groups based on serum 11dhTXB2/creatinine values of 2,524 and 675.1 pg/mg, respectively. **Table 1** shows the basic characteristics of the patients. Age, gender, platelet count, thrombocytocrit, the numbers of patients with hypertension, diabetes mellitus, coronary heart disease, the plasma total cholesterol, total triglyceride, LDL-C, HDL-C levels of the patients did not significantly differ between the groups (**Table 1**). The NHISS scores were significantly higher in the aspirin resistance, than in the aspirin sensitive group, and plasma HDL-C levels were notably higher in the aspirin sensitive group, than in the aspirin resistance group (**Table 1**).

**Table 1:** Characteristics of study subjects.

	Aspirin sensitive	Aspirin resistance	p
11dhTXB2/creatinine (pg/mg)	675.1 ± 29.53	2524 ± 231.6	**<0.0001**
Age (years)	62.58 ± 1.205	62.73 ± 1.412	0.9695
Gender(male/female)	70/28	41/10	0.2106
Smoking, n (%)	0.357	0.327	0.7113
Hypertension, n (%)	0.734	0.711	0.762
Diabetes mellitus, n (%)	0.252	0.288	0.6602
Coronary artery diseases, n (%)	0.285	0.231	0.4689
Platelet counts (*10^9^/L)	201.2 ± 6.234	212.6 ± 7.518	0.5396
Thrombocycrit (%)	0.2152 ± 0.0829	0.1856 ± 0.12460	0.1194
TG (mmol/L)	1.486 ± 0.09800	1.680 ± 0.1704	0.1326
TC (mmol/L)	4.280 ± 0.09733	4.345 ± 0.1504	0.3215
LDL-C (mmol/L)	2.662 ± 0.08846	2.753 ± 0.1214	0.0833
HDL-C (mmol/L)	1.096 ± 0.04277	1.078 ± 0.03721	**0.0298**
NIHSS	2.929 ± 0.2941	3.140 ± 0.4227	0.6252

TG, total triglyceride; TC, total cholesterol; LDL-C, low-density lipoprotein cholesterol; HDL-C, high-density lipoprotein cholesterol; NIHSS: National Institutes Health Stoke Scale. Bold values means the result is of statistical significance.

### Plasma H19 Level Is Closely Associated With Aspirin Resistance

**Figure 1:**
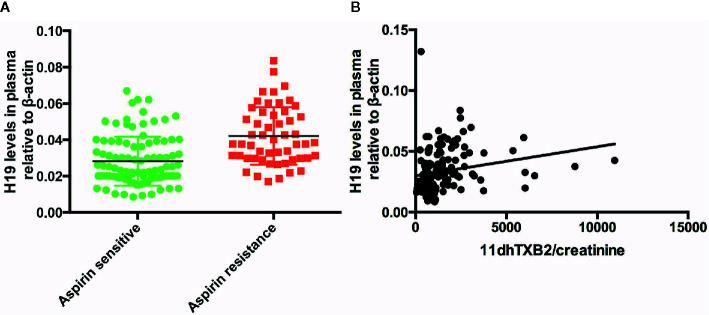
The expression of H19 in the patients and the association between H19 and urine 11dhTXB2/creatinine. **(A)** The comparison of H19 between aspirin sensitive and aspirin resistance group. **(B)** Plasma H19 level is positively associated with the value of urine 11dhTXB2/creatinine (R = 0.04364, p = 0.0106).

We measured the plasma levels of H19 in all the patients to determine whether it participates in the regulation of AR. We found that plasma H19 levels were significantly higher in in the AR, than in the AS group (p = 0.0203, **Figure 1A**). and that the urinary 11dhTXB2/creatinine levels and plasma H19 levels were positively associated (R = 0.04364, p = 0.0106; **Figure 1B**).

### Plasma H19 Level Is Positively Correlated With the Level of 8-Iso-prostaglandin-2α

**Figure 2:**
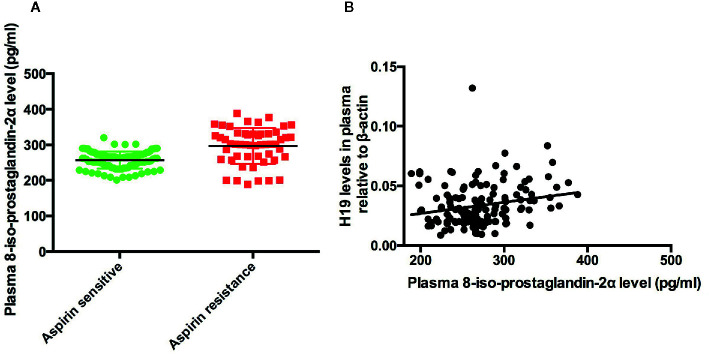
The expression of 8-iso-PGF2α in the patients and the association between H19 and 8-iso-PGF2α. **(A)** The comparison of 8-iso-PGF2α between aspirin sensitive and aspirin resistance group. **(B)** Plasma H19 level is positively associated with 8-iso-PGF2α (R = 0.04561, p = 0.0089).

To elucidate the mechanism of H19 induced aspirin resistance, we tested plasma level of 8-iso-prostaglandin-2α in aspirin sensitive and aspirin resistance patients and analyzed the correlation between H19 and 8-iso-PGF2α. We found that plasma 8-iso-PGF2α level in aspirin resistant patient was higher than that of aspirin sensitive group (p < 0.0001, **Figure 2A**). And plasma H19 was positively associated with the level of 8-iso-PGF2α (R=0.04561, p=0.0089, **Figure 2B**).

### H19 Could Serve as a Serological Biomarker and Independent Risk Factor for Aspirin Resistance

**Figure 3:**
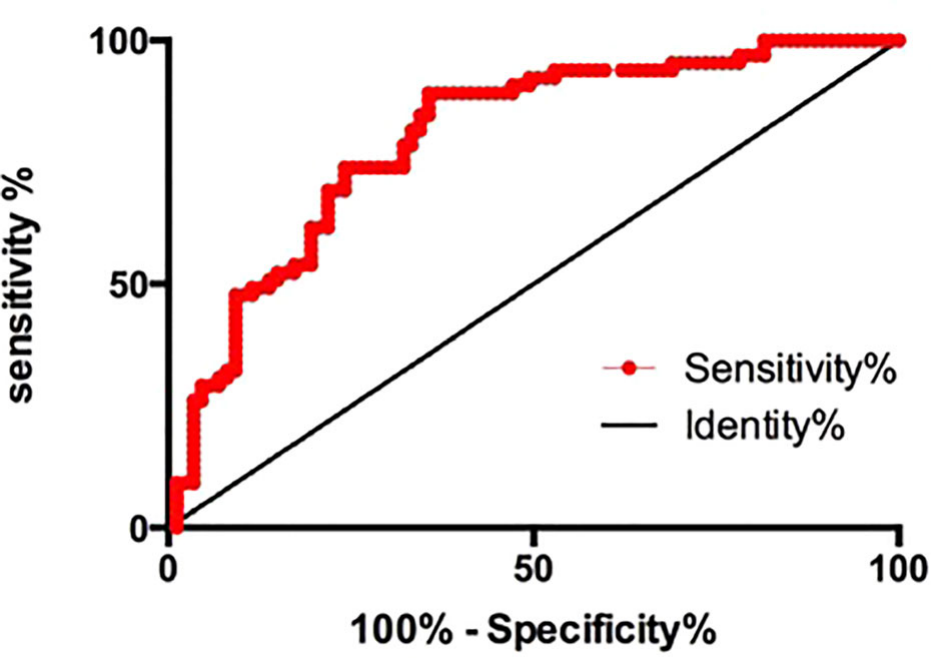
The receiver operating characteristic curve of H19 for the prediction of aspirin resistance. Area under curve, 0.8005; 95% CI, 0.7301–0.8710; p<0.0001; specificity=75.86207%; sensitivity=73.84615%.

We investigated whether H19 could diagnose AR using ROC curves. H19 was highly sensitive and specific with regard to diagnosing AR (area under curve, 0.8005; 95% CI, 0.7301–0.8710; p<0.0001; specificity = 75.86207%; sensitivity = 73.84615%; **Figure 3**). Multiple logistic regression analysis then determined that H19 is an independent risk factor for AR (**Table 2**, OR=1.129, p=0.0321). Levels of HDL-C were notably higher in the aspirin sensitive group than in the aspirin resistance group (**Table 1**, p=0.0298), but HDL-C could not independently influence the effects of aspirin in patients (**Table 2**, OR=0.584, p=0.1426).

**Table 2:** The determination of independent risk factors for aspirin resistance.

	OR	95% CI	p
H19	1.129	1.016–1.201	**0.0321**
HDL-C	0.584	0.235–1.025	0.1426

H19, long non coding RNA H19; HDL-C, high-density lipoprotein cholesterol. Bold values means the result is of statistical significance.

### Aspirin Resistance and Plasma H19 Are Associated With Cerebral Ischemic Stroke Recurrence

**Figure 4:**
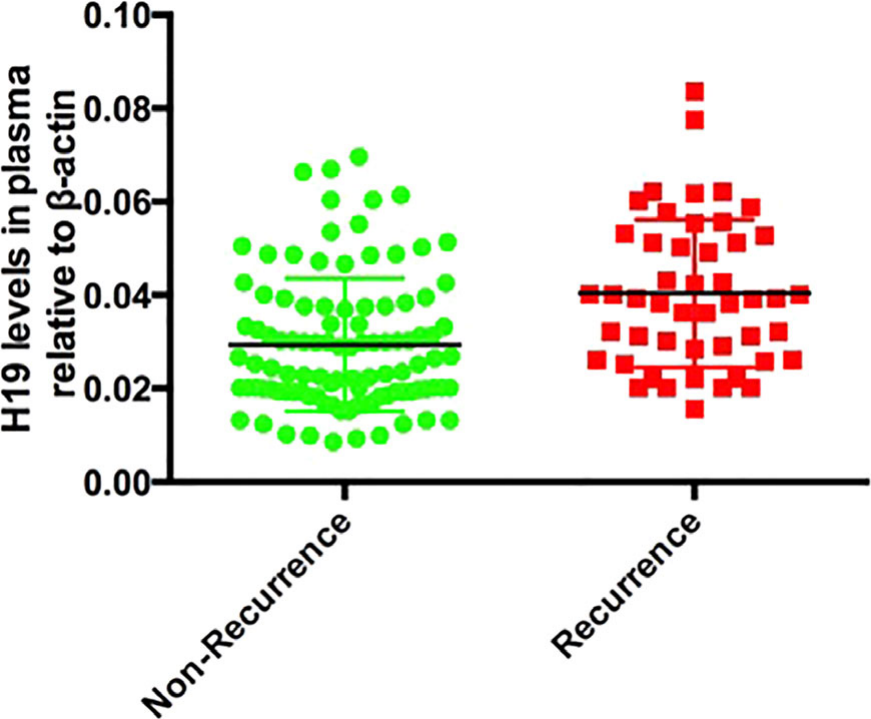
The comparison of H19 between recurrent stroke and non-recurrent stroke patients, p < 0.0001.

We followed up all 150 patients for 1 year to assess the relationship between aspirin resistance and recurrent cerebral ischemic stroke. We separated the patients into recurrence and non-recurrence group based on the follow-up data. We found that 28 and 24 patients in the Recurrence group and Non-Recurrence group respectively, had AR, and the difference was significant (p<0.0001; **Table 3**). We then analyzed the plasma H19 levels in the Recurrence group and Non-Recurrence group and found that plasma H19 was significantly upregulated in the R group (p < 0.0001; **Figure 4**).

**Table 3:** The relationship between aspirin resistance and stroke recurrence.

	Recurrence	Non-Recurrence	p
Aspirin sensitive	20	78	
Aspirin resistance	28	24	**<0.0001**

Bold values means the result is of statistical significance.

## Discussion

Orally administration of low-dose (100–200 mg) aspirin is the most common and important means of preventing and treating cerebral ischemic stroke (Powers et al., 2018). However, recent studies have found that aspirin might not always exert maximal anti-platelet effects. This is due to aspirin resistance, which affects 5% to 45% of the population (Mason Peter et al., 2005). While the exact mechanism of aspirin resistance is unclear.

Long non-coding RNA H19 (H19) is one of the first long non-coding RNAs to be discovered. The expression of H19 can be upregulated by hypoxia. We previously revealed that H19 expression functions in cerebral ischemia through activating neuronal autophagy and promoting microglial M1 phenotype polarization (Wang et al., 2017a; Wang et al., 2017b). H19 also prevents the neurogenesis process after an ischemic stroke event (Wang et al., 2019). We also found that a H19 gene polymorphism could affect the susceptibility to cerebral ischemic stroke (Wang et al., 2017a). Plasma H19 levels are negatively associated with the prognosis of stroke (Wang et al., 2019). Since aspirin resistance decreases the ability of aspirin to prevent and treat cerebral ischemic stroke, susceptibility to stroke increases and worsens the prognosis. We hypothesized that H19 is involved in AR.

Aspirin resistance is mainly determined by evaluating platelet function and measuring TXA2 levels. Platelet function evaluations are complex and require methods such as light projection aggregation assays, platelet function analyzers and rapid platelet function assay (Stephanie and Rohit, 2008). Measurements of platelet functions and TXA2 levels in blood require invasive sampling. Here, we measured the levels of 11dhTXB2 to reflect the extent of aspirin resistance, because it is the metabolic product of TXA2, and can be tested in urine. Urinary 11dhTXB2/creatinine values greater than 1,500 pg/mg indicated aspirin resistance. Urine samples can be collected non-invasively and 11dhTXA2 levels can be measured in urine using ELISA kits. Thus, quantifying the urinary 11dhTXB2/creatinine levels has become a popular index of aspirin resistance (Lopez et al., 2014).

We studied the relationship between H19 expression and aspirin resistance in 150 patients with cerebral ischemic stroke. We assigned them to the aspirin resistance and aspirin sensitive groups based on their urinary values for 11dhTXA2/creatinine. The level of H19 was markedly higher in the aspirin resistance, than in the aspirin sensitive group, and it was positively associated with the urinary 11dhTXA2/creatinine levels. We also determined that H19 is an independent risk factor for ischemic stroke and that it could diagnose aspirin resistance with high specificity and sensitivity. Therefore, the plasma H19 levels might serve as a serological marker of aspirin resistance.

8-iso-PGF2 is a rich F2 iso-prostaglandin existed in human body with high bioactivity. It could react with thromboxane A2/prostaglandin H2 receptor in platelet, blood vessel and artherosclerotic plaque to regulate platelet function or induce the contraction of the blood vessels. It has been reported that the overload of oxidative stress lead to the production of 8-iso-PGF2 and cause the biosynthesis of TXA2 and finally induce aspirin resistance (Cipollone et al., 2000). We proved in this study that the level of 8-iso-PGF2 was significantly higher in aspirin resistance group and H19 expression levels were positively associated with that of 8-iso-PGF2, which indicated that H19 might induce aspirin resistance through regulate the production of 8-iso-PGF2.

Many studies have shown that aspirin resistance directly lead to recurrent cerebral ischemic stroke, and such recurrence has even been viewed as an objective reflection of aspirin resistance (Zhang et al., 2017). We confirmed that aspirin resistance is closely associated with recurrent ischemic stroke. When patients were divided into the Recurrence and Non-Recurrence group, the plasma levels of H19 is significantly higher in the Recurrence group than the Non-Recurrence group. These results helped explain the findings of our previous research in which higher plasma level of H19 values resulted in the slower recovery of the neurological defects. Since almost all the patients consume aspirin after an ischemic stroke event, those with high plasma H19 levels might have had aspirin resistance and finally shown a bad prognosis or ischemic stroke recurrence.

Plasma levels of HDL-C were significantly higher in the aspirin sensitive group than that of aspirin resistance group. The previous studies have demonstrated that levels of HDL-C and total cholesterol/HDL-C are closely associated with the occurrence of aspirin resistance (Kang et al., 2008; Ertugrul et al., 2010). In contrast, herein, we found that total cholesterol/HDL-C levels did not significantly differ between these groups and that a low HDL-C level is not an independent risk factor for aspirin resistance. Further studies are needed to elucidate the relationship between HDL-C levels and aspirin resistance.

In conclusion, we revealed that H19 is closely associated with aspirin resistance and it may induce aspirin resistance through regulating 8-iso-PGF2. H19 might be a serological marker for the diagnosis of aspirin resistance with high specificity and sensitivity. This study provides a deeper understanding of the mechanism underlying aspirin resistance and strengthens the clinical value of H19 for the diagnosis and treatment of ischemic stroke. However, our study has some limitations. The number of patients included in this study is limited; hence, multicenter clinical studies are required to confirm our results. Further, the mechanism through which H19 induces AR also needs to be investigated.

## Data Availability Statement

The raw data supporting the conclusions of this article will be made available by the authors, without undue reservation.

## Ethics Statement

The studies involving human participants were reviewed and approved by the Ethic Committee of Shengjing Hospital and China Medical University. The patients/participants provided their written informed consent to participate in this study. Written informed consent was obtained from the individual(s) for the publication of any potentially identifiable images or data included in this article.

## Author Contributions

JW: responsible for the design and carry out of the experiment and the writing of the manuscript. BC, YG, DH, HZ: responsible for the carry out of the experiment. YC, YL: responsible for the check of the English writing and structure of the manuscript. JF, YXG: responsible for the check of the design and writing of the manuscript.

## Funding

This work was supported by the National Natural Science Foundation of China (Grant No. 81801171; 2018); the National Natural Science Foundation of China (Grant No. 81771271; 2017); the Natural Science Foundation Guidance Plan of Liaoning Province (Grant No. 20180550707; 2018); the China Postdoctoral Science Foundation (Grant No. 2018M641742; 2018); the Natural Science Foundation Guidance Plan of Liaoning Province (Grant No. 20170541053; 2017).

## Conflict of Interest

The authors declare that the research was conducted in the absence of any commercial or financial relationships that could be construed as a potential conflict of interest

The reviewer LL declared a shared affiliation, with no collaboration, with several of the authors, HZ, YL, to the handling editor at the time of review.
